# Spontaneous Evolution in Bilirubin Levels Predicts Liver-Related Mortality in Patients with Alcoholic Hepatitis

**DOI:** 10.1371/journal.pone.0100870

**Published:** 2014-07-11

**Authors:** Minjong Lee, Won Kim, Yunhee Choi, Sunhee Kim, Donghee Kim, Su Jong Yu, Jeong-Hoon Lee, Hwi Young Kim, Yong Jin Jung, Byeong Gwan Kim, Yoon Jun Kim, Jung-Hwan Yoon, Kook Lae Lee, Hyo-Suk Lee

**Affiliations:** 1 Department of Internal Medicine and Liver Research Institute, Seoul National University College of Medicine, Seoul, Korea; 2 Department of Internal Medicine, Seoul Metropolitan Government Seoul National University Boramae Medical Center, Seoul, Korea; 3 Medical Research Collaborating Center, Seoul National University Hospital, Seoul, Korea; 4 Department of Internal Medicine, Gangnam Healthcare Center, Seoul National University Hospital, Seoul, Korea; Sezione di Gastroenterologia, Italy

## Abstract

The accurate prognostic stratification of alcoholic hepatitis (AH) is essential for individualized therapeutic decisions. The aim of this study was to develop a new prognostic model to predict liver-related mortality in Asian AH patients. We conducted a hospital-based, retrospective cohort study using 308 patients with AH between 1999 and 2011 (a derivation cohort) and 106 patients with AH between 2005 and 2012 (a validation cohort). The Cox proportional hazards model was constructed to select significant predictors of liver-related death from the derivation cohort. A new prognostic model was internally validated using a bootstrap sampling method. The discriminative performance of this new model was compared with those of other prognostic models using a concordance index in the validation cohort. Bilirubin, prothrombin time, creatinine, potassium at admission, and a spontaneous change in bilirubin levels from day 0 to day 7 (SCBL) were incorporated into a model for AH to grade the severity in an Asian patient cohort (MAGIC). For risk stratification, four risk groups were identified with cutoff scores of 29, 37, and 46 based on the different survival probabilities (*P*<0.001). In addition, MAGIC showed better discriminative performance for liver-related mortality than any other scoring system in the validation cohort. MAGIC can accurately predict liver-related mortality in Asian patients hospitalized for AH. Therefore, SCBL may help us decide whether patients with AH urgently require corticosteroid treatment.

## Introduction

Alcoholic liver disease (ALD) is one of the major causes of end-stage liver disease throughout the world [1]. With increasing alcohol consumption, ALD has become a significant global health burden [2]. ALD has a broad disease spectrum that encompasses simple steatosis, steatohepatitis, cirrhosis, and hepatocellular carcinoma (HCC) [3]. Of these diseases, alcoholic hepatitis (AH) is histologically characterized by steatosis, hepatocellular inflammation, necrosis, and pericellular fibrosis [4]. Patients with severe AH have a reported 30-day mortality of up to 50% [5]–[8].

Given the conspicuous short-term mortality, there is a stringent necessity for early recognition of patients who suffer from severe AH to select the appropriate management. The Glasgow alcoholic hepatitis score (GAHS) [5], model for end-stage liver disease (MELD) [9], age, serum bilirubin, international normalized ratio of prothrombin time, and serum creatinine (ABIC) [10], Maddrey’s discriminant function (MDF) [11], an early change in bilirubin levels (ECBL) [6], and the Lille model [12] are the prevailing prognostic scoring systems used to predict survival in AH patients.

However, the aforementioned scoring systems have several inherent limitations because each system has been validated using data from the regional Western cohort, and the sample size of each cohort was not large enough to extrapolate each system to other ethnicities [5], [6], [9]–[14]. In addition, concern about heterogeneity in the study population may be raised because the previous studies included severe AH patients treated with corticosteroids as well as patients who were untreated due to mild or moderate severity [6], [9], [10], [12].

The pathogenesis of AH involves a multi-factorial process, including the metabolism of alcohol to toxic products, Kupffer cell stimulation by endotoxins, and nutritional impairment leading to liver injury and inflammation [15]. The risk for hepatocellular injury related to heavy drinking is significantly associated with race and ethnicity [16]. Although the prevalence of heavy drinking among various racial and ethnic groups is similar [17], Mexican and non-Hispanic Black American drinkers are at higher risk of liver-related death than Caucasian drinkers [16].

In particular, in the metabolic pathway of alcohol degradation, aldehyde dehydrogenase (ALDH) and cytochrome P4502E1 (CYP4502E1) play key roles in the breakdown of acetaldehyde to acetic acid [18], [19]. An *ALDH2* allele, ALDH2*2 produces various toxins and inflammatory cytokines due to its insufficient degradation of acetaldehyde [18], [19]. The ALDH2*2 allele is exclusively expressed in the Asian population, although its prevalence varies across Asian ethnicities [20].

In this context, whether existing prognostic scoring systems could accurately predict the natural outcomes of untreated patients with AH in other ethnic populations should be further validated. Despite the racial disparity involved in the metabolism of alcohol and the cultural diversity regarding alcohol consumption [20], few studies have attempted to externally validate the prognostic scoring systems developed in Western populations, particularly for Asian patients with AH. Herein, we attempted to validate the aforementioned scoring systems in Asian AH patients and to develop a new prognostic scoring model that stratifies the risk of liver-related mortality in Asian populations.

## Materials and Methods

### Study Population

This analysis incorporated two data cohorts, a derivation cohort and a validation cohort, in a predictive survival model. The derivation cohort consisted of AH patients fulfilling the eligibility criteria at Seoul Metropolitan Government Seoul National University Boramae Medical Center (SMG-SNU BMC) between December 1999 and June 2011. The validation cohort was used to externally validate the predictive survival model constructed from the derivation cohort. The validation cohort consisted of AH patients fulfilling the eligibility criteria at Seoul National University Hospital (SNUH) between January 2005 and June 2011.

All of the potential candidates were meticulously reviewed, and the demographic, clinical, and laboratory data were extracted from the electronic medical records of both university-affiliated hospitals. The presence of acute AH was confirmed by clinical and laboratory criteria as follows: (i) alcohol consumption within 2 months and exceeding 60 g/day for males and 40 g/day for females, (ii) rapid deterioration of liver function during the past 2 months, (iii) an aspartate/alanine aminotransferase (AST/ALT) ratio greater than 2 with an AST level greater than 45 IU/L and less than 300 IU/L [21]–[23], and (iv) a total bilirubin level greater than 2 mg/dL [9]. The following patients were excluded: (i) those with liver disease causes other than alcohol consumption (i.e., viral hepatitis, autoimmune hepatitis, and drug-induced liver injury) or HCC; (ii) those who died from non-liver-related causes, such as non-liver malignancies, cardiovascular events, or unknown reasons; (iii) those who exhibited uncontrolled infection or recent gastrointestinal bleeding within 15 days; and (iv) those receiving pentoxifylline and/or corticosteroids. This study was approved by the Institutional Review Boards of both SMG-SNU BMC and SNUH.

### Data Collection and Analysis

The first available laboratory data within 24 hours from admission were used to calculate the baseline MELD, GAHS, ABIC, MDF, and Child-Turcotte-Pugh (CTP) scores. To calculate the dynamic change of existing scoring systems and a spontaneous change in bilirubin levels (SCBL), we collected available laboratory data at day 7 after admission. Liver function-related symptoms, such as ascites and hepatic encephalopathy, were evaluated to calculate CTP scores at admission. The presence of ascites was confirmed by radiologic evaluation, such as ultrasonography or computed tomography. Hepatic encephalopathy was indicated by the presence of altered mentality and flapping tremor during a physical examination. The primary endpoint was liver-related death. Causes of death were verified by electronic medical records or National Death Registry data (http://www.kostat.go.kr/).

### Statistical Analysis

Demographic, clinical, and biochemical characteristics and clinical outcomes were summarized using the median [1^st^ quartile, 3^rd^ quartile] for continuous variables and frequencies (proportion) for categorical variables. The Cox proportional hazards model was applied to develop a predictive survival model for patients with AH from the derivation cohort. The non-linear effects and proportional hazards assumption of continuous variables were investigated using restricted cubic splines [24]. Variables showing significant non-linear effects, such as serum creatinine and prothrombin time, were logarithmically transformed. Variables reaching a *P*-value<0.2 in univariate analyses were included in the multivariate analysis. The final model was chosen on the basis of the clinical and statistical significance. A new prognostic scoring system obtained from the Cox model was validated using a concordance index, Uno’s C-index [25]. Its 95% confidence interval (CI) was calculated by bootstrap estimation using 10,000 bootstrap re-samples. The discriminative performance of the survival function of the new prognostic scoring system was compared with those of other prognostic scoring systems according to Uno’s C-indices in the validation cohort. Based on the new scoring system, risk stratification was performed using unbiased recursive partitioning [26], which determined the optimal cutoff scores to discriminate AH patients according to their survival. Significant cutoff scores were decided at the 5% level of significance. Kaplan-Meier survival analysis was performed to compare cumulative survival probabilities according to the cutoff scores. Statistical analyses were performed using R software version 2.13.2 (R Foundation for statistical computing, Vienna, Austria, http://www.r-project.org/).

## Results

### Baseline Characteristics of the Study Population

**Figure 1:**
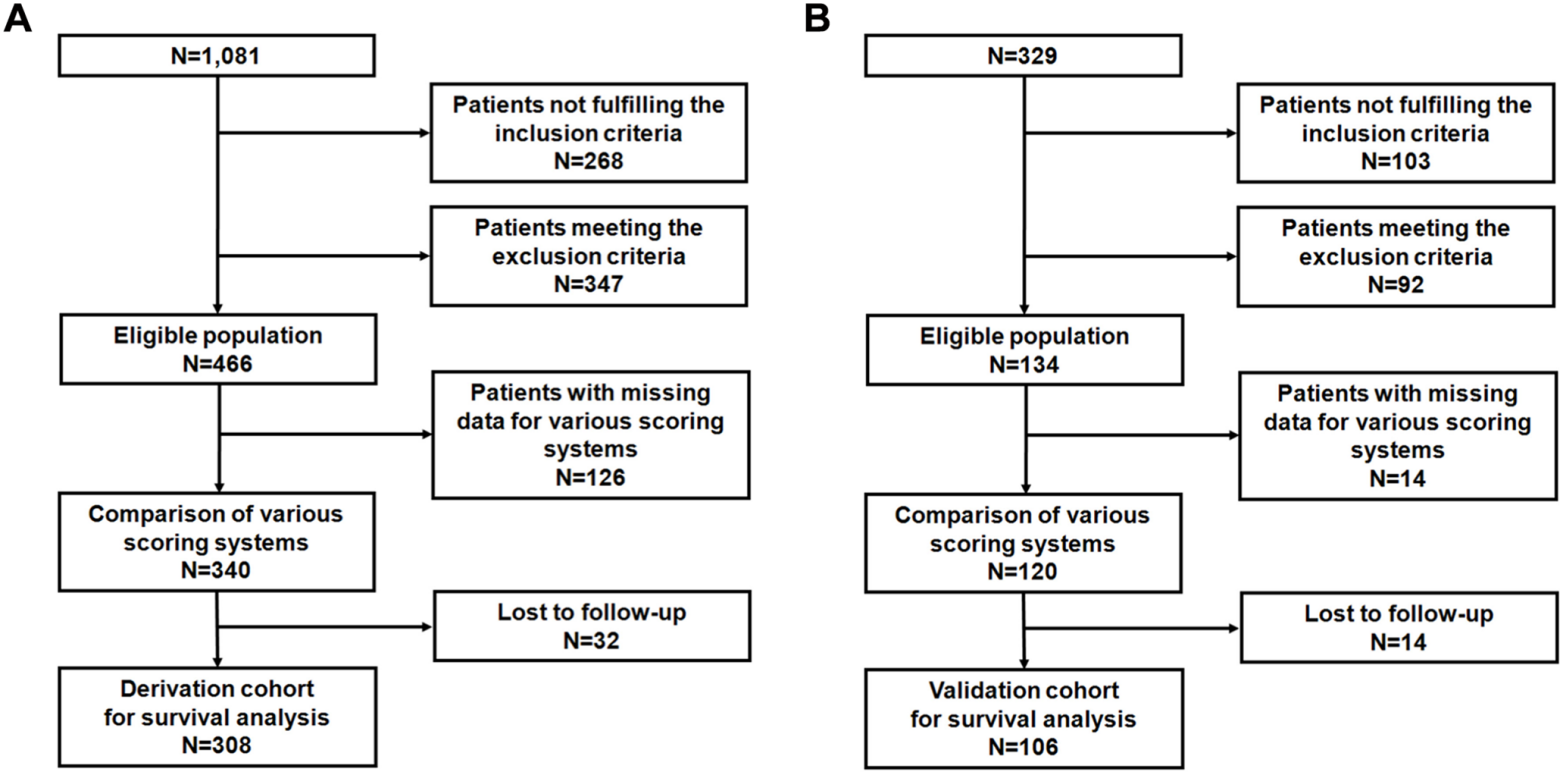
Flow charts for the derivation cohort (A) and the validation cohort (B).

A total of 1,410 consecutive inpatients were diagnosed with AH on the basis of the International Classification of Diseases, 10th Revision (ICD-10), at two referral hospitals. Of all the patients, 600 fulfilled the eligibility criteria for survival analysis to predict liver-related death. Patients treated with corticosteroids or pentoxifylline were excluded; 82 patients in the derivation cohort and 23 patients in the validation cohort. After excluding patients with missing laboratory data for all of the prognostic scoring systems, 460 patients were selected. Then, 46 patients were excluded because of the lack of available data on survival at discharge or death dates. Finally, 414 patients were recruited for survival analysis to predict liver-related death; 308 patients from SMG-SNU BMC were included in the derivation cohort (Fig. 1A), and 106 patients from SNUH were included in the validation cohort (Fig. 1B).

Table 1 summarizes the baseline characteristics of the study population. The male preponderance (86.2%) was a remarkable finding. All of the patients were of Asian ethnicity. The median bilirubin levels in the derivation and validation cohorts were 5.2 and 6.0 mg/dL, respectively. The median AST and ALT levels were 134 and 45.5 IU/L, respectively, in the derivation cohort and 104 and 39 IU/L in the validation cohort. The median MELD score was 17.0 in the derivation cohort and 20.7 in the validation cohort. The median MDF was 29.0 in the derivation cohort and 49.1 in the validation cohort. Of the 414 included patients, 295 (71.3%) presented at least one feature of hepatic decompensation, including ascites and hepatic encephalopathy. The main causes of liver-related death in patients with AH included hepatic failure, infection, gastrointestinal bleeding, and hepatorenal syndrome.

**Table 1:** Baseline Demographic, Clinical, and Biochemical Characteristics of the Study Population.

	Total population (n = 414)	Derivation cohort (n = 308)	Validation cohort (n = 106)	*P* value
Age (yr)	51 [45, 59]	51 [45, 58]	54 [44, 60]	0.128†
Gender (%)				0.021‡
Male	357 (86.2)	273 (88.6)	84 (79.3)	
Female	57 (13.8)	35 (11.4)	22 (20.8)	
Alcohol intake (g/day)	113 [56.6, 150]	100[60, 141.3]	113 [56.5, 150]	0.363†
Liver-related mortality				
30-day mortality (%)	61 (14.7)			0.222‡
90-day mortality (%)	77 (18.6)			0.092‡
Variables at admission				
WBC, ×10^3^/µL	8.3 [6.0,1.2]	7.9 [6.0, 11.1]	9.2 [6.0,11.3]	0.288†
ANC, ×10^3^/µL	6.1 [3.9, 9.5]	6.0 [4.0, 9.4]	6.7 [3.9, 9.7]	0.866†
Platelet, ×10^3^/µL	94.5 [64, 150]	91.5 [63, 149.5]	102.5 [70, 150.1]	0.647†
Hemoglobin, g/dL	10.8 [8.8, 12.5]	11.0 [8.8, 12.7]	10.3 [8.7,11.9]	0.244*
PT INR	1.5 [1.3, 1.9]	1.4 [1.3, 1.8]	1.7 [1.4, 2.1]	<0.001†
Albumin, g/dL	2.7 [2.4, 3.1]	2.7 [2.5, 3.1]	2.6 [2.2, 2.9]	<0.001†
AST, IU/L	126.5 [79, 200]	134 [84.5, 210.5]	104 [68, 184]	0.002†
ALT, IU/L	44 [27, 70]	45.5 [28, 74]	39 [21, 61]	0.007†
Total bilirubin, mg/dL	5.5 [3.1, 11.4]	5.2 [2.9, 9.8]	6.0 [3.3, 17.7]	0.049†
ALP, IU/L	142 [107, 201]	145 [109, 206]	131.5 [98, 179]	0.023†
Cholesterol, mg/dL	118 [87, 156]	120 [94, 159]	104 [79, 149]	0.016†
BUN, mg/dL	13 [8], [26]	13 [8], [24]	16 [9], [28]	0.231†
Creatinine, mg/dL	1.0 [0.8, 1.3]	0.9 [0.7, 1.3]	1.0 [0.9, 1.7]	0.008†
Na, mmol/L	134.3 [129, 137.9]	134.3 [129.0, 137.6]	135.0 [130, 138]	0.942†
K, mmol/L	3.9 [3.4, 4.5]	3.8 [3.3, 4.4]	4.1 [3.5, 4.6]	0.004†
SCBL, mg/dL	−1 [−2.4, 0.3]	−1.1 [−2.3, 0.1]	−0.8 [−2.7, 0.4]	0.892†
Prognostic Scores				
MELD	17.9 [13.1, 23.1]	17.0 [12.8, 21.5]	20.7 [15.4, 26.2]	<0.001†
GAHS	7 [6], [8]	7 [6], [8]	8 [7], [9]	<0.001†
ABIC	7.5 [6.6, 8.6]	7.3 [6.5, 8.3]	7.9 [7], [9]	<0.001†
MDF	33.6 [19.9, 57.8]	29.0 [18.0, 51.4]	49.1 [32.4, 72.0]	<0.001†
CTP	10 [9], [12]	10 [8], [11]	11 [9], [12]	0.002†
Initial symptoms at admission (%)				
Ascites	269 (65.0)	194 (63.0)	75 (70.8)	0.111‡
Hepatic encephalopathy	89 (21.5)	64 (20.8)	25 (23.6)	0.443‡
Causes of liver-related death (%)				0.792§
Infection	30 (37.0)	26 (38.9)	4 (28.6)	
Bleeding	24 (29.5)	19 (28.3)	5 (35.7)	
Hepatic failure	16 (19.9)	14 (20.8)	2 (14.3)	
Hepatorenal syndrome	11 (13.6)	8 (12.0)	3 (21.4)	

Values are expressed as the median and interquartile range (IQR).

WBC, white blood cell; ANC, absolute neutrophil count; PT INR, international normalized ratio of prothrombin time; AST, aspartate aminotransferase; ALT, alanine aminotransferase; ALP, alkaline phosphatase; BUN, blood urea nitrogen; Na, sodium; K, potassium; SCBL, spontaneous change in total bilirubin levels; MELD, model for end-stage liver disease; GAHS, Glasgow alcoholic hepatitis score; ABIC, age, serum bilirubin, INR, and serum creatinine; MDF, Maddrey’s discriminant function; CTP, Child-Turcotte-Pugh.

*Independent T-Test.

† Wilcoxon Rank Sum Test.

‡ Chi-Square Test.

§ Fisher’s Exact Test.

### Development of a New Prognostic Scoring Model for Asian Populations

We assessed potential risk factors among the biochemical variables reflecting hematologic status, liver function, and kidney function. Table 2 depicts the variables significantly correlated with liver-related mortality in the univariate and multivariate analyses, which included all of the parameters that comprise the MELD, GAHS, ABIC, MDF, CTP, and the dynamic change in bilirubin levels. The initial clinical symptoms at presentation were excluded from the final Cox model because they were incapable of being incorporated into an objective scoring system. Five variables were statistically significant in the Cox regression model and independently predicted liver-related mortality: bilirubin (*P*<0.001), prothrombin time (*P*<0.001), creatinine (*P*<0.001), and potassium (*P* = 0.0153) at day 0 and the difference in serum bilirubin levels from day 0 to day 7 (*P* = 0.001) (Table 2). Based on the multivariate analysis, a model for alcoholic hepatitis to grade the severity in an Asian patient cohort (MAGIC) score can be calculated using the formula presented in Table 3.

**Table 2:** Factors Predicting Survival in the Derivation Cohort.

Variable	Univariate		Multivariate	
	Hazard Ratio (95% CI)	*P* Value	Hazard Ratio (95% CI)	*P* Value
Age	1.015 (0.995, 1.035)	0.1519		
Gender	1.017 (0.507, 2.038)	0.9624		
WBC, ×10^3^/µL	1.026 (0.999, 1.055)	0.0632		
ANC, ×10^3^/µL	1.032 (1.003, 1.062)	0.0311		
Hemoglobin, g/dL	0.862 (0.796, 0.933)	0.0003		
Platelet, ×10^3^/µL	0.676 (0.475, 0.963)	0.0302		
Cholesterol, mg/dL	0.991 (0.986, 0.996)	0.0002		
Albumin, g/dL	0.329 (0.199, 0.546)	<0.0001		
Total bilirubin, mg/dL	1.058 (1.033, 1.083)	<0.0001	10.913 (3.649, 32.639)	<0.001
AST, IU/L	0.999 (0.997, 1.001)	0.3411		
ALT, IU/L	0.994 (0.988, 0.999)	0.031		
ALP, IU/L	0.998 (0.995, 1.001)	0.1519		
ln(PT INR)	12.35 (7.034, 21.68)	<0.0001	1.049 (1.011, 1.089)	<0.001
BUN, mg/dL	1.019 (1.013, 1.024)	<0.0001		
ln(Creatinine), mg/dL	3.353(2.545, 4.417)	<0.0001	2.540 (1.757, 3.671)	<0.001
Na, mmol/L	0.953 (0.925, 0.981)	0.0012		
K, mmol/L	1.908 (1.530, 2.380)	<0.0001	1.394 (1.090, 1.784)	0.015
SCBL, mg/dL	1.115 (1.072, 1.160)	<0.0001	1.067 (1.028, 1.108)	0.001

WBC, white blood cell; ANC, absolute neutrophil count; AST, aspartate aminotransferase; ALT, alanine aminotransferase; ALP. alkaline phosphatase; PT INR, international normalized ratio of prothrombin time; BUN, blood urea nitrogen; Na, sodium; K, potassium; SCBL, spontaneous change in total bilirubin levels; MAGIC, model for alcoholic hepatitis to grade severity in an Asian patient cohort.

**Table 3:** Equation for MAGIC.

MAGIC Score
In h(t, x) = ln h_0_(t) + 2.8007×ln(PT INR) + 0.9321×ln(creatinine) + 0.3325×potassium + 0.0651×SCBL+ 0.0826×total bilirubin at day 0 – 0.0856×total bilirubin at day 0×ln(PT INR)

PT INR, international normalized ratio of prothrombin time; SCBL, spontaneous change in total bilirubin levels; MAGIC, model for alcoholic hepatitis to grade severity in an Asian patient cohort.

### Validation of Prognostic Scoring Systems in an Asian Population

For internal validation, we compared prognostic accuracy in terms of the prediction of liver-related death between MAGIC and five other scoring systems (Table 4). The c-statistics for discrimination of the survival model in the derivation cohort were as follows: MAGIC, 0.858 (95% CI: 0.815–0.899); MELD, 0.827 (95% CI, 0.783–0.869); GAHS, 0.754 (95% CI, 0.702–0.805); ABIC, 0.756 (95% CI, 0.699–0.809); MDF, 0.758 (95% CI, 0.696–0.815); and CTP, 0.693 (95% CI, 0.619–0.759). The c-statistics of dynamic changes in MELD, ABIC, GAHS, and MDF between days 0 and 7 were also compared with that of MAGIC: 0.552 (95% CI, 0.468–0.638) for Δ MELD; 0.582 (95% CI, 0.496–0.670) for Δ ABIC; 0.477 (95% CI, 0.401–0.555) for Δ GAHS; and 0.632 (95% CI, 0.546–0.716) for Δ MDF. Taken together, MAGIC showed the best prognostic performance among all the scoring systems in predicting liver-related death.

**Table 4:** Internal Validation of MAGIC in the Derivation Cohort (n = 308).

Scoring system	Concordance index (95% CI)
MAGIC	0.858 (0.815, 0.899)
MELD	0.827 (0.783, 0.869)
GAHS	0.754 (0.702, 0.805)
ABIC	0.756 (0.699, 0.809)
MDF	0.758 (0.696, 0.815)
CTP	0.693 (0.619, 0.759)
Δ MELD	0.552 (0.468, 0.638),
Δ GAHS	0.477 (0.401, 0.555)
Δ ABIC	0.582 (0.496, 0.670)
Δ MDF	0.632 (0.546, 0.716)

MELD, model for end-stage liver disease; GAHS, Glasgow alcoholic hepatitis score; ABIC, age, serum bilirubin, INR, and serum creatinine; MDF, Maddrey’s discriminant function; CTP, Child-Turcotte-Pugh.

The symbol ‘Δ’ indicates the change in each score from day 0 to day 7.

MAGIC was also externally validated and compared with other scoring systems in the validation cohort. The c-statistics in the validation cohort were as follows: MAGIC, 0.871 (95% CI, 0.788–0.939); MELD, 0.784 (95% CI, 0.667–0.890); GAHS, 0.765 (95% CI, 0.650–0.865); ABIC, 0.846 (95% CI, 0.746–0.928); MDF, 0.811 (95% CI, 0.724–0.890); and CTP, 0.640 (95% CI, 0.495–0.771). The c-statistics of dynamic changes in MELD, ABIC, GAHS, and MDF between days 0 and 7 were as follows: Δ MELD, 0.734 (95% CI, 0.581–0.861); Δ ABIC, 0.713 (95% CI, 0.511–0.874); Δ GAHS, 0.520 (95% CI, 0.316–0.706); and Δ MDF, 0.648 (95% CI, 0.404–0.842) (Table 5). Collectively, MAGIC also showed the best prognostic performance in predicting liver-related mortality in the validation cohort.

**Table 5:** External Validation of MAGIC in the Validation Cohort (n = 106).

Scoring system	Concordance index (95% CI)
MAGIC	0.871 (0.788, 0.939)
MELD	0.784 (0.667, 0.890)
GAHS	0.765 (0.650, 0.865)
ABIC	0.846 (0.746, 0.928)
MDF	0.811 (0.724, 0.890)
CTP	0.640 (0.495, 0.771)
Δ MELD	0.734 (0.581, 0.861)
Δ GAHS	0.520 (0.316, 0.706)
Δ ABIC	0.713 (0.511, 0.874)
Δ MDF	0.648 (0.404, 0.842)

MAGIC, model for alcoholic hepatitis to grade the severity in an Asian patient cohort; MELD, model for end-stage liver disease; GAHS, Glasgow alcoholic hepatitis score; ABIC, age, serum bilirubin, INR, and serum creatinine; MDF, Maddrey’s discriminant function; CTP, Child-Turcotte-Pugh.

The symbol ‘Δ’ indicates the change in each score from day 0 to day 7.

### Risk Stratification by the MAGIC Score in terms of Liver-related Death

**Figure 2:**
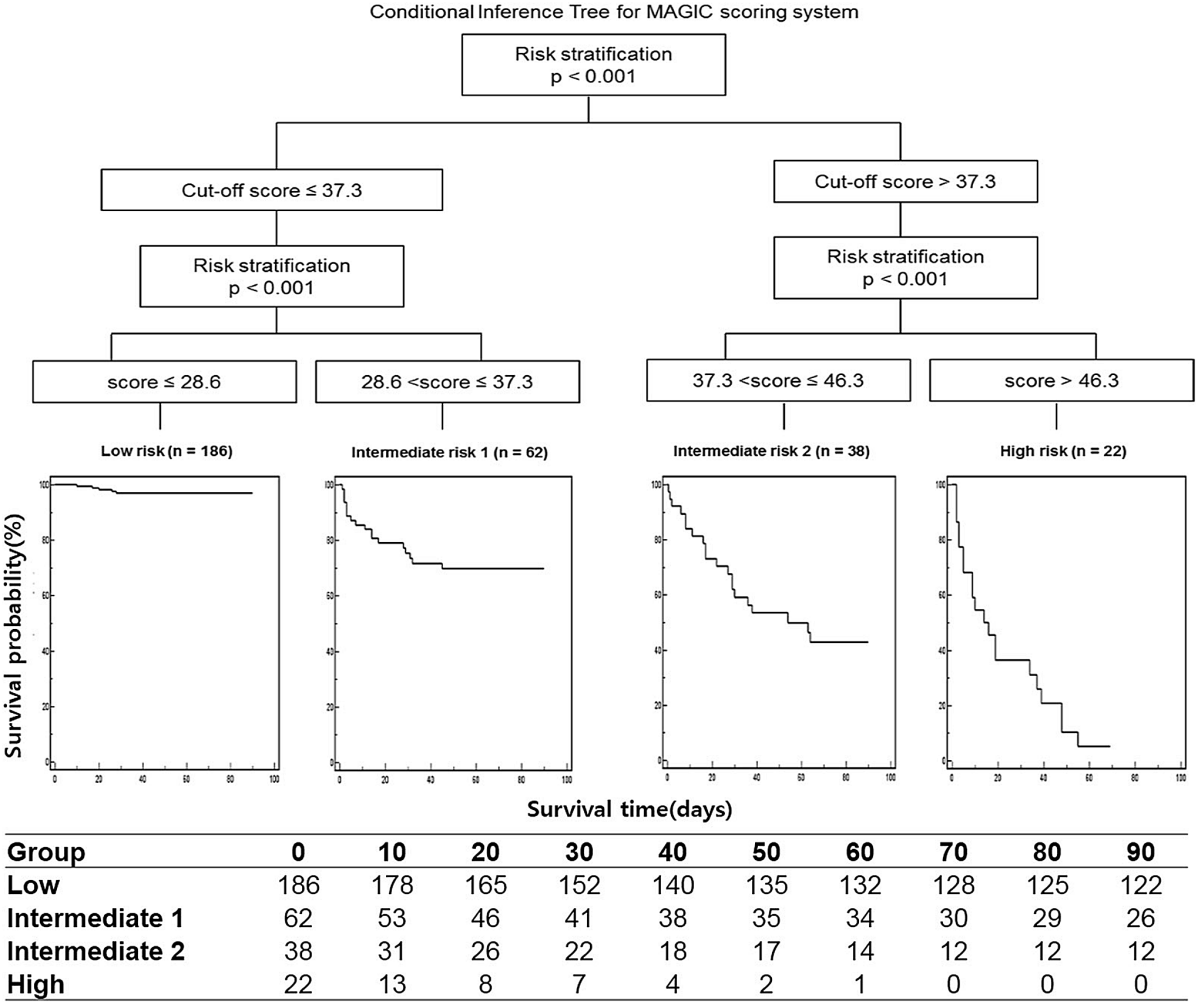
Risk stratification of liver-related death according to the cumulative survival probabilities in the derivation cohort. We analyzed survival data using conditional inference trees to stratify a derivation cohort into four risk groups and to determine the optimal cutoff scores of MAGIC. The X-axis indicates the survival time (days), and the Y-axis indicates the survival probability (%). MAGIC, model for alcoholic hepatitis to grade the severity in an Asian patient cohort.

**Figure 3:**
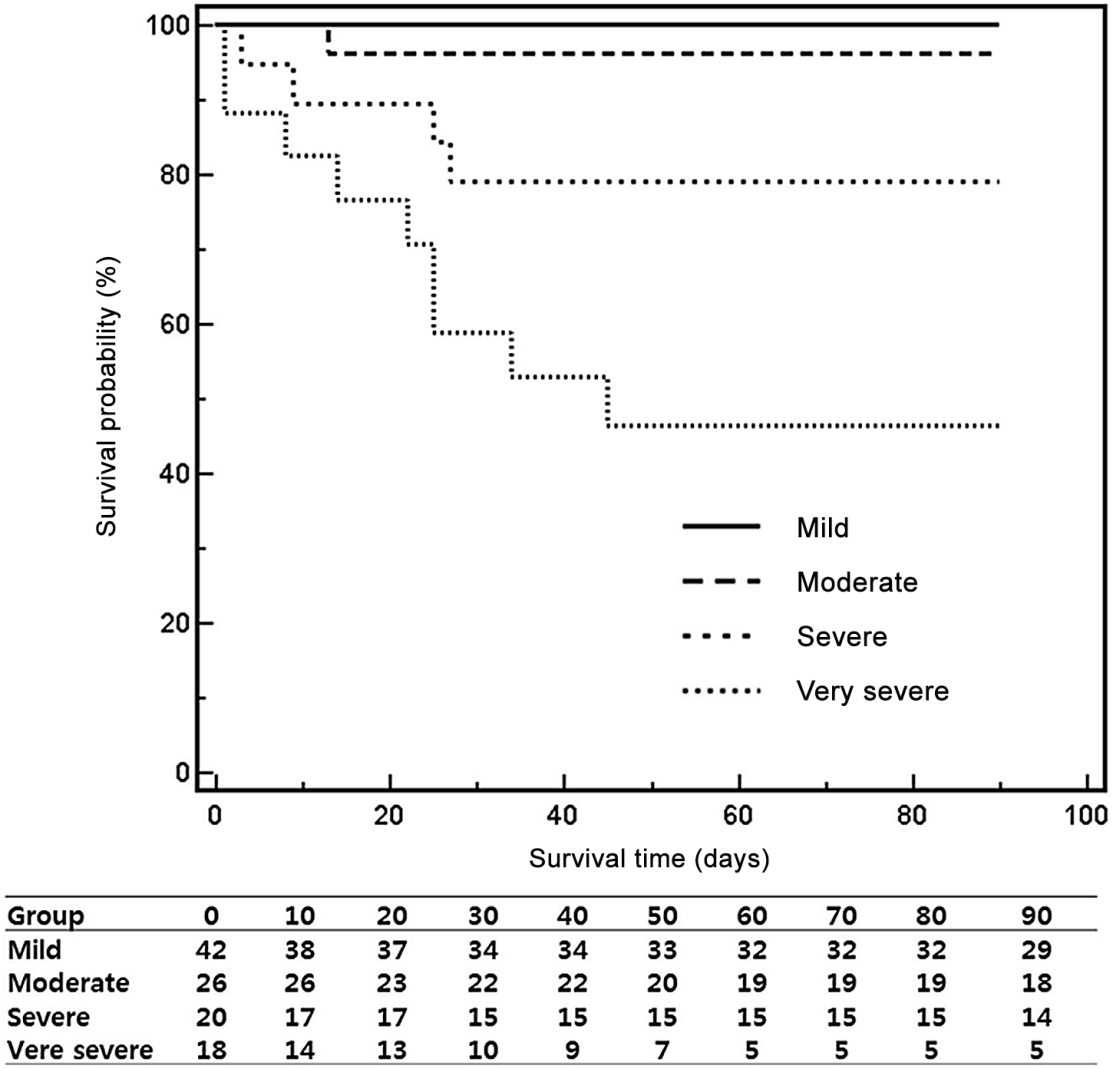
Validation of the optimal cutoff scores of MAGIC for individual risk groups of liver-related death in the validation cohort. The X-axis indicates the survival time (days), and the Y-axis indicates the survival probability (%). The solid line represents the mild-risk group (≤29, n = 42), the dashed line represents the moderate-risk group (29<risk score≤37, n = 26), the large dotted line represents the severe-risk group (37<risk score≤46, n = 20), and the small dotted line represents the very severe-risk group (>46, n = 18). MAGIC, model for alcoholic hepatitis to grade the severity in an Asian patient cohort.

The derivation cohort was stratified into four risk subgroups according to the survival probabilities, and the optimal MAGIC cutoff scores were chosen based on the conditional inference trees: mild risk, ≤29; moderate risk, 29< risk score≤37; severe risk, 37< risk score≤46; and very severe risk, >46 (*P*<0.001, Fig. 2). Risk stratification using the MAGIC score was also verified in the validation cohort. The optimal MAGIC cutoff scores significantly stratified the validation cohort into four risk subgroups (*P*<0.001 by the log-rank test, Fig. 3). The MAGIC cutoff scores also stratified the total study population into four risk subgroups, and the 90-day cumulative survival probabilities in individual risk groups were as follows: mild risk, 97.8%; moderate risk, 78.4%; severe risk, 58.6%; and very severe risk, 27.5% (Table S1). The median scores of the prognostic scoring systems (MELD, ABIC, GAHS, and MDF) for individual risk groups are presented in Table S1.

## Discussion

In this retrospective evaluation of an Asian population with AH, MAGIC efficiently allowed us to identify those with diverse prognoses; some patients with good prognoses could be managed with supportive treatment, and others at high risk of liver-related death could be potential candidates for specific treatment such as corticosteroids or pentoxifylline. Thus, the MAGIC score may play an important role in accurately predicting the outcomes of hospitalized patients with AH.

In the current study, SCBL independently predicted liver-related death in a multivariate analysis after adjusting for bilirubin, prothrombin time, albumin, creatinine, sodium, and potassium. SCBL was chosen as one of the potential predictors of liver-related death in this new model for two reasons. First, it has been proven that a dynamic change in bilirubin levels, such as an ECBL or the Lille model, predicts survival outcomes in severe AH patients treated with corticosteroids by assessing the early on-treatment response [6], [12]. However, the dynamic changes in prothrombin time, creatinine, and potassium were not considered as potential candidates because these factors are subject to common treatment measures including transfusion and fluid therapy. Second, we set an endpoint for SCBL of “seven” days after admission prior to starting specific treatment because the median time to starting corticosteroid therapy for patients with severe AH was approximately 7 days after admission [27].

Our findings demonstrated that hyperkalemia might be significantly associated with an increased risk of liver-related mortality. Hyperkalemia may worsen with the progressive deterioration of AH because of metabolic acidosis, kidney injury, and brain edema. Although hyponatremia was a strong predictor of mortality in patients with decompensated cirrhosis (chronic liver failure) [28], [29], hyperkalemia might be clinically more relevant to patients with severe AH (acute liver injury or acute-on-chronic liver failure) than hyponatremia.

Of the parameters in MAGIC, each factor may signal two different types of systemic complications of AH, either renal or hepatic impairment. A more prolonged prothrombin time, higher bilirubin levels, and a greater SCBL may reflect a more severe degree of acute hepatic failure. High potassium and creatinine levels may indicate acute kidney injury in AH patients. From these standpoints, the pattern of MAGIC that reflected the worsening of hepatic and renal function was in line with the patterns of other prognostic scoring systems.

In the current study, we validated MELD, ABIC, GAHS, and MDF in an Asian population with AH and compared them with MAGIC in terms of the prediction of liver-related mortality. To date, no concrete data are available regarding the external validation of Western scoring systems in Asian populations. Here, the c-statistic values of conventional scoring systems, when applied to Asian patients with AH, were similar to those in Western populations. However, the dynamic changes of the aforementioned scoring systems showed poorer prognostic performances than those calculated using only baseline variables, in agreement with results from previous studies [9], [12]. The Lille model, one of the most important scoring systems reflecting the dynamic change of liver injury after corticosteroid treatment, was not analyzed in the current study because we excluded AH patients treated with pentoxifylline and/or corticosteroids to avoid the influence of specific treatment on survival outcomes.

Our study has some intrinsic limitations. First, because our study was not a prospectively designed clinical trial, liver biopsies were unfortunately not routinely performed to diagnose AH. We relied on the clinical diagnosis of AH, which is more feasible in a community-based, large-scale study. There have been many debates regarding whether liver biopsy is required to diagnose AH because of its low yield (30%) and the rare availability of the transjugular approach [30]. Second, some discrepancies in disease severity existed between the derivation cohort and the validation cohort. It might be unrealistic to match all the laboratory findings in one cohort to those in the other cohort. Because the severity of AH based on MELD, GAHS, ABIC, and MDF was higher in the validation cohort than in the derivation cohort, MAGIC showed the superior prognostic performance in the validation cohort than in the derivation cohort. The external validation of MAGIC in other populations may also be needed in the future. Finally, the MAGIC score included the bilirubin levels at day 7, which were not known at admission. Thus, there may be some difficulties in guiding the rapid initiation of corticosteroid treatment because the MAGIC score cannot be calculated immediately at admission. However, it typically takes at least 7 days to exclude the possibility of systemic infections by culture studies and to confirm the histological diagnosis of AH prior to starting corticosteroids [27].

With these caveats in mind, the unique features of MAGIC are as follows: (i) MAGIC was derived from Asian AH patients and compared with Western prognostic scoring systems, (ii) it focused on the prediction of the natural outcomes of severe AH patients not treated with pentoxifylline and/or corticosteroids, (iii) it emphasized the prognostic role of hyperkalemia for AH mortality, and (iv) the spontaneous evolution of liver injury was incorporated into the new model.

In conclusion, the MAGIC score, an easily used risk calculator, permits clinicians to assess the individual risk of liver-related death from severe AH in Asian populations. Given the validation of prognostic scoring systems, MAGIC was the best-fitting model for AH in our Asian AH cohort. Taken together, MAGIC as well as Western prognostic models can be applied to Asian AH patients for risk stratification. Patients with a severe or very severe risk of death (MAGIC score>37) could benefit from more aggressive management, such as liver transplantation. In the future, further prospective studies should be implemented for the external validation of our new model in other populations.

## Supporting Information

Table S1 **Baseline Demographic, Clinical and Biochemical Characteristics of the Total Population and Individual Risk Groups.**
(DOCX)
