# Correction: Spontaneous Evolution in Bilirubin Levels Predicts Liver-Related Mortality in Patients with Alcoholic Hepatitis

**DOI:** 10.1371/journal.pone.0167184

**Published:** 2016-11-18

**Authors:** Minjong Lee, Won Kim, Yunhee Choi, Sunhee Kim, Donghee Kim, Su Jong Yu, Jeong-Hoon Lee, Hwi Young Kim, Yong Jin Jung, Byeong Gwan Kim, Yoon Jun Kim, Jung-Hwan Yoon, Kook Lae Lee, Hyo-Suk Lee

The equation in Table 3 is incorrect. Please see the corrected Table 3 here.

**Table 3:** Equation for MAGIC.

MAGIC Score = 10×{2.8007 × ln(PT INR) + 0.9321 × ln(creatinine) + 0.3325 × potassium + 0.0651 × SCBL+ 0.0826 × total bilirubin at day 0–0.0856 × total bilirubin at day 0 × ln(PT INR)}

PT INR, international normalized; SCBL, spontaneous change in bilirubin levels from day 0 to day 7; MAGIC, model for alcoholic hepatitis to grade severity in an Asian patient cohort.
