# Supplementary material for: Spontaneous Evolution in Bilirubin Levels Predicts Liver-Related Mortality in Patients with Alcoholic Hepatitis
Source: PLoS One. 2014 Jul 11;9(7):e100870. doi: 10.1371/journal.pone.0100870 (PMC4094461; doi:10.1371/journal.pone.0100870)
Supplement: Table S1 — Baseline Demographic, Clinical and Biochemical Characteristics of the Total Population and Individual Risk Groups. (DOCX) [file pone.0100870.s001.docx]

Supplementary Table 1. Baseline Demographic, Clinical and Biochemical Characteristics of the Total Population and Individual Risk Groups

|  | Total population (n=414) | Mild risk (n=228) | Moderate risk (n=88) | Severe risk (n=58) | Very severe risk (n=40) | *P* Value |  |
| --- | --- | --- | --- | --- | --- | --- | --- |
| Age (yr) | 51 [45, 58] | 50.5 [44, 59] | 52 [45, 59] | 52 [43, 56] | 54.5 [48, 58.5] | 0.240† |  |
| Gender (%) |  |  |  |  |  | 0.962‡ |  |
| Male | 357 (86.2) | 195 (85.5) | 76 (86.4) | 51 (87.9) | 35 (87.5) |  |  |
| Female | 57 (13.8) | 33 (14.5) | 12 (13.6) | 7 (12.1) | 5 (12.5) |  |  |
| Alcohol intake (g/day) | 113 [60,150] | 113 [56.5,150] | 113 [60,141.3] | 113 [70.6,141.3] | 113 [60,150] | 0.949† |  |
| Liver-related mortality (%) |  |  |  |  |  |  |  |
| 30-day mortality | 61 (14.7) | 5 (2.2) | 16 (18.2) | 19 (32.8) | 21 (52.5) | <0.001‡ |  |
| 90-day mortality | 77 (18.6) | 5 (2.2) | 19 (21.6) | 24 (41.4) | 29 (72.5) | <0.001‡ |  |
| Variables at admission |  |  |  |  |  |  |  |
| WBC, ×10^3^/μL | 8.2 [6.0, 11.7] | 7.6 [5.7, 10.6] | 8.3 [5.5, 12.9] | 10.7 [7.3, 14.2] | 9.5 [7.4, 15.1] | <0.001† |  |
| ANC, ×10^3^/μL | 6.0 [3.9, 9.5] | 5.4 [3.5, 8.0] | 5.7 [3.5, 10.4] | 8.7 [5.8, 12.5] | 8.0 [6.0, 12.5] | <0.001† |  |
| Hemoglobin, g/dL | 10.8 [8.8, 12.5] | 11.5 [9.8, 13.0] | 9.9 [8, 11.8] | 9.7 [7.7,11.7] | 8.8 [7.8, 10.8] | <0.001† |  |
| Platelet, ×10^3^/μL | 94 [64, 149] | 104 [70, 160] | 88.5 [56.5, 142.5] | 103.5 [76, 146] | 73.5 [48.5, 107.5] | 0.004† |  |
| PT INR | 1.5 [1.3, 1.9] | 1.3 [1.2, 1.5] | 1.7 [1.5, 1.9] | 2 [1.7, 2.5] | 2.4 [2.1, 3.2] | <0.001† |  |
| Total bilirubin, mg/dL | 5.1 [3, 10.4] | 4.2 [2.7, 7.1] | 5.8 [3.2, 12.3] | 13.2 [5.6, 22.2] | 13.3 [5.9, 25.7] | <0.001† |  |
| Albumin, g/dL | 2.7 [2.4, 3.1] | 2.9 [2.6, 3.2] | 2.7 [2.5, 2.9] | 2.4 [2.1, 2.6] | 2.3 [2.1, 2.6] | <0.001† |  |
| Cholesterol, mg/dL | 116 [86, 156] | 137 [105, 183] | 105 [81, 138] | 101 [75, 130] | 77.5 [52, 91.5] | <0.001† |  |
| AST, IU/L | 125 [79, 200] | 138.5 [90.5, 222] | 104 [63.5, 190] | 118 [74, 161] | 108 [71, 184.5] | 0.01† |  |
| ALT, IU/L | 43 [27, 70] | 51 [28, 82] | 34.5 [25.5, 52] | 36.5 [23, 59] | 43.5 [25.5, 61] | <0.001† |  |
| ALP, IU/L | 139 [104, 199] | 158 [120, 219] | 131 [100, 177] | 123 [96, 198] | 117.5 [83.5,173.5] | <0.001† |  |
| BUN, mg/dL | 14 [8, 25] | 10 [7, 16] | 17.5 [10, 29] | 21 [14, 42] | 47 [25.5, 68.5] | <0.001† |  |
| Creatinine, mg/dL | 1 [0.8, 1.3] | 0.8 [0.7, 1] | 1.1 [0.9, 1.5] | 1.7 [1, 2.3] | 2.9 [1.6, 3.8] | <0.001† |  |
| Na, mmol/L | 134.3 [129.6, 138] | 135.4 [132, 138] | 135 [131.1, 138] | 130.7 [124.9, 135] | 128.4 [120.4, 133] | <0.001† |  |
| K, mmol/L | 3.9 [3.4, 4.5] | 3.7 [3.2, 4.2] | 4 [3.5, 4.6] | 4.2 [3.7, 4.8] | 5.2 [4.1, 6.1] | <0.001† |  |
| SCBL | -1 [-2.4, 0.3] | -1.4 [-2.6, -0.5] | -0.7 [-2, 0.9] | -0.6 [-4.2, 1.4] | 1.6 [-0.4, 7] | <0.001† |  |
| Prognostic scoring system |  |  |  |  |  |  |  |
| MELD | 17.8 [13.1, 23] | 13.4 [11, 16.7] | 20.3 [18.8, 22.4] | 27.0 [23.5, 33.1] | 35.3 [29.5, 39.8] | <0.001† | |
| GAHS | 7 [6, 8] | 6 [6, 7] | 8 [7, 8] | 9 [8, 9] | 10 [9, 10] | <0.001† | |
| ABIC | 7.4 [6.6, 8.5] | 6.8 [ 6.2, 7.7] | 7.6 [7, 8.6] | 8.2 [7.7, 9.1] | 9.8 [8.8, 10.8] | <0.001† | |
| MDF | 33.4 [19.9, 56.8] | 22.6 [12.5, 32.9] | 45.8 [32.7, 57.7] | 66.0 [52.2, 86.7] | 89.3 [65.9, 101.6] | <0.001† | |
| CTP | 10 [8, 12] | 9 [8, 10] | 11 [10, 12] | 12 [10, 13] | 12 [11, 13] | <0.001† | |

Values are expressed as the median and interquartile range (IQR).

WBC, white blood cell; ANC, absolute neutrophil count; PT INR, international normalized ratio of prothrombin time; AST, aspartate aminotransferase; ALT, alanine aminotransferase; ALP, alkaline phosphatase; BUN, blood urea nitrogen; Na, sodium; K, potassium; SCBL, spontaneous change in total bilirubin levels; MELD, model for end-stage liver disease; GAHS, Glasgow alcoholic hepatitis score; ABIC, age, serum bilirubin, INR, and serum creatinine; MDF, Maddrey’s discriminant function; CTP, Child-Turcotte-Pugh.

†Kruskal-Wallis Test

‡Chi-Square Test
